# Comparison of the Confluence-Initiated Neurogenic Differentiation Tendency of Adipose-Derived and Bone Marrow-Derived Mesenchymal Stem Cells

**DOI:** 10.3390/biomedicines9111503

**Published:** 2021-10-20

**Authors:** Szu-Hsien Wu, Yu-Ting Liao, Chi-Han Huang, Yi-Chou Chen, En-Rung Chiang, Jung-Pan Wang

**Affiliations:** 1 Department of Surgery, Taipei Veterans General Hospital, Taipei 112, Taiwan; shwu3@vghtpe.gov.tw (S.-H.W.); a98811008@gmail.com (C.-H.H.); 2 Department of Surgery, School of Medicine, National Yang Ming Chiao Tung University, Taipei 112, Taiwan; rondoo0614@gmail.com; 3 Division of Plastic and Reconstructive Surgery, Department of Surgery, School of Medicine, National Defense Medical Center, Taipei 112, Taiwan; 4 Department of Orthopaedics & Traumatology, Taipei Veterans General Hospital, Taipei 112, Taiwan; grammyaward_3@hotmail.com; 5 Department of Orthopedics, Taoyuan General Hospital, Ministry of Health and Welfare, Taoyuan 330, Taiwan; secretdovac@gmail.com

**Keywords:** adipose-derived mesenchymal stem cells (ADSCs), bone marrow-derived mesenchymal stem cells (BMSCs), confluence-initiated differentiation, neurogenic differentiation

## Abstract

Adipose-derived mesenchymal stem cells (ADSCs), which tended to neurogenically differentiate spontaneously after achieving high confluence, were observed. Human ADSCs reaching 80% confluence were cultured in DMEM without an inducing factor for 24 h and then maintained in DMEM plus 1% FBS medium for 7 days. The neurogenic, adipogenic, and osteogenic genes of the factor-induced and confluence-initiated differentiation of the ADSCs and bone marrow-derived mesenchymal stem cells (BMSCs) at passages 3 to 5 were determined and compared using RT-qPCR, and the neurogenic differentiation was confirmed using immunofluorescent staining. In vitro tests revealed that the RNA and protein expression of neuronal markers, including class III β-tubulin (*TUBB3*), microtubule-associated protein 2 (*MAP2*), neurofilament medium polypeptide (*NEFM*), neurofilament heavy polypeptide (*NEFH*), and neurofilament light polypeptide (*NEFL*), had been enhanced in the confluence-initiated differentiation of the ADSCs. In addition, the expressions of neurotrophins, such as the nerve growth factor (*NGF*), brain-derived neurotrophic factor (*BDNF*), and glial cell-derived neurotrophic factor (*GDNF*), were also elevated in the confluence-initiated differentiation of the ADSCs. However, the confluent ADSCs did not show a tendency toward spontaneous adipogenic and osteogenic differentiation. Moreover, compared with the confluent ADSCs, the tendency of spontaneous neurogenic, adipogenic, and osteogenic differentiation of the confluent human bone marrow mesenchymal stem cells (BMSCs) was not observed. The results indicated that ADSCs had the potential to spontaneously differentiate into neuron-like cells during the confluent culture period; however, this tendency was not observed in BMSCs.

## 1. Introduction

Over the past two decades, mesenchymal stem cells (MSCs) have been studied extensively. Owning the abilities of self-renewal and multi-lineage differentiation [1,2], MSCs are considered to be a promising therapeutic treatment for organ repair and regeneration. MSCs are known to be isolated from several organs, such as bone marrow [3,4], adipose tissues [5], dental tissues [6], umbilical cord blood [7], and the liver [8]. Adipose-derived mesenchymal stem cells (ADSCs), which can be gained from adipose tissue, are easily and numerously available and can be provided as an alternative for tissue engineering and cell therapy [9,10]. ADSCs have been described to possess morphological, phenotypic, and functional characteristics like bone marrow-derived mesenchymal stem cells (BMSCs), and they can differentiate into adipocytes [2], osteocytes, and chondrocytes under different protocols [11]. ADSCs are beneficial to wound healing [12] and multiple tissue regeneration [13], and protect against senescence [14]. The abilities of neurogenic differentiation of BMSCs [15,16,17] and ADSCs [18,19,20] have been discussed in several papers. BMSCs have demonstrated promising results in peripheral nerve regeneration studies [21]. Artificial nerve conduits injected with BMSCs have been found to regenerate nerves [22,23,24]. Applications of ADSCs on advancing nerve regeneration in animal models have also been adequately reported [25,26,27,28,29]. ADSCs can differentiate into Schwann cell (SCs)-like [30] and neuron-like [31] phenotypes in vitro and in vivo to benefit nerve regeneration. Moreover, ADSCs can secrete neurotrophic factors to facilitate intrinsic healing by host SCs [32,33], such as the vascular endothelial growth factor (VEGF), brain-derived growth factor (BDNF), nerve growth factor (NGF), and glial line-derived neurotrophic factor (GDNF). The comparisons of the neurogenic differentiation capacity of BMSCs and ADSCs were studied, and the results revealed that the ability of ADSCs was better than that of BMSCs [34,35]. 

Cell confluence, which is an important factor of in vitro expansion, influences the proliferation, biological properties, and osteogenic differentiation of BMSCs [36]. The proliferation of ADSCs from low density was higher than that from high density [37]. Cultivated cell density may cause changes in gene expression, proliferation rates, and cell morphology in BMSCs [38]. There were more genes associated with angiogenesis, proliferation, migration, survival, anti-inflammatory effects, and differentiation detected in confluent ADSCs [39]. This suggested that the confluence of an MSC harvest might be an influential issue for clinical treatments.

In this study, human ADSCs, which had reached high confluence, at passages 3 to 5 tended to neurogenically differentiate spontaneously, but did not exhibit adipogenic and osteogenic differentiation. A high confluence culture may play an important role in spontaneously inducing the neurogenic differentiation of ADSCs. The spontaneous neurogenic differentiation potential of ADSCs without an external neurogenic factor-induced medium needs to be elucidated. We aimed to compare the potential of the neurogenic differentiation of ADSCs with or without a factor-inducing medium. To determine spontaneous adipogenic and osteogenic differentiation, the spontaneous differentiation potentials of ADSCs were compared with BMSCs.

## 2. Materials and Methods

### 2.1. Expansion of BMSCs and ADSCs

The human BMSC cell line was obtained from Professor Shih-Chieh Hung’s Lab [40]. The human ADSCs (*n* = 3) were isolated from fat tissues of healthy female donors undergoing liposuction of the abdomen, and the surgery procedures were approved by the Institutional Review Board (IRB) of the Taipei Veterans General Hospital. Stromal vascular fraction (SVF) isolation was carried out according to previous studies [41,42]. Briefly, the oil layer, aqueous layer, and excess blood were removed through centrifugation and washed with Hank’s Balanced Salt Solution (HBSS; Gibco, Carlsbad, CA, USA). Fat was mixed with 0.075% (*w*/*v*) collagenase (Sigma-Aldrich, St. Louis, MO, USA) in a prewarmed HBSS buffer at the ratio of 1:1 or 2:1, and was incubated at 37 °C for 30 min in a shaking water bath. Half of the volume of Dulbecco’s Modified Eagle Medium (DMEM; Gibco, Grand Island, NY, USA) with 10% fetal bovine serum (FBS; Invitrogen, Carlsbad, CA, USA) was added for neutralization and then washed with PBS (Gibco/BRL, Grand Island, NY, USA). After a series of filtrations with 100-μm, 70-μm (optional), and 40-μm filters, 4–7 × 10^5^ living cells in 1 mL of the normal human fat tissue could be achieved. The MSC immunophenotypic characteristics, including CD11b, CD14, CD19, CD34, CD45, CD79a, HLA-DR, CD29, CD44, CD73, CD90, and CD105, of the isolated human ADSCs were confirmed using the FACS Canto II Cytometer System running Diva software (Becton Dickinson, San Jose, CA, USA). All the methods were carried out in accordance with relevant guidelines and regulations, and all the adipose tissue was collected with the patients’ informed consent.

About 2–3 × 10^6^ cells were seeded and cultured in DMEM containing 10% FBS and antibiotic–antimitotic solution (Corning life science, New York, NY, USA) for the cell culture. The fresh growth medium was changed every two days, and the cells were propagated every four days at a 1:5 split before the growth reached 80% of the confluence.

### 2.2. Confluence-Initiated Differentiation

For spontaneous differentiation, the 80% confluent cells at passages 5–6 were cultured in a culture medium without the inducing factor for 24 h. The cells were then maintained in the DMEM plus 1% FBS medium for seven days.

### 2.3. Factor-Induced Neurogenic Differentiation

For the factor-induced neurogenic differentiation procedure, cells with 80% confluence at passages 3–5 were induced by a culture medium supplemented with 50 μM. All-trans-retinoic acid (RA; Sigma-Aldrich) and 1 mM 2-Mercaptoethanol (β-ME; Sigma-Aldrich), which were placed in a 37 °C, 5% CO_2_, and 20% O_2_ incubator for 24 h. Then, the differentiated cells were maintained with the DMEM containing 1% FBS for seven days [43].

### 2.4. Osteogenic Induction Medium (OIM)-Induced Differentiation

The OIM included a culture medium supplemented with 10% FBS, 50 μg/mL ascorbic acid-2 phosphate (Nacalai, Kyoto, Japan), 0.01 μM dexamethasone (Sigma-Aldrich), and 1 mM b-glycerol phosphate (Sigma-Aldrich), all of which were used to induce osteogenic differentiation. The medium was changed every two days for a period of seven days.

### 2.5. Adipogenic Induction Medium (AIM)-Induced Differentiation

The AIM comprised a culture medium supplemented with 10% FBS, 50 μg/mL ascorbic acid-2 phosphate, 0.1 μM dexamethasone, 50 μM indomethacin (Sigma-Aldrich), 45 μM 3-isobutyl-1-methylxanthine (Sigma-Aldrich), and 1 μg/mL insulin (Sigma-Aldrich), all of which were used to induce adipogenic differentiation. The medium was changed every two days for a period of seven days.

### 2.6. RT-qPCR Analysis

Total RNA was extracted using the Trizol reagent (Invitrogen). For cDNA synthesis, reverse transcription reactions and synthesis were carried out by the iScript^TM^ cDNA Synthesis Kit (Bio-Rad^®^, Hercules, CA, USA). The cDNA was synthesized by the M-MuLV reverse transcriptase, and PCR was performed with specific primers and the Fast SYBR^®^ Green Master Mix (Applied Biosystems, Foster City, CA, USA). All the sequences of the primers are listed in Table 1. Each cycle consisted of the following steps: the initial denaturation step for 10 min at 95 °C, denaturation for 3 s at 95 °C, and annealing for 30 s at 60 °C (40 cycles). Glyceraldehyde-3-phosphate dehydrogenase (*GAPDH*) was detected as an internal control. The results were analyzed using the software supplied with a machine utilizing the comparative C_T_ (ΔΔC_T_) method.

### 2.7. Immunofluorescence (IF) Staining and Intensity Quantification

Primary antibodies against microtubule-associated protein 2 (MAP2; GeneTex Inc., Irvine, CA, USA), class III β-tubulin (TUBB3; Santa Cruz Biotechnology, Santa Cruz, CA, USA), neurofilament medium polypeptide (NEFM; OriGene Technologies, Medical Center Drive, Rockville, MD, USA), and neurofilament light polypeptide (NEFL; OriGene Technologies) were added on the slides at an appropriate dilution for IF staining. The slides were then incubated with the secondary antibodies with green fluorescence protein against the primary antibodies for fluorescence analysis. All the slides were counterstained with 4,6-diamidino-2-phenylindole (DAPI; Sigma-Aldrich) for nuclear (blue) fluorescent staining. Total florescence intensity in one field was measured by the Image-Pro Plus (v4.5.0.29, Media Cybernetics, Silver Spring, MD, USA), and the average intensity from 120 to 140 cells in six fields was calculated for each slide. The average florescence intensities of individual cells in the images were also calculated.

### 2.8. Statistical Analysis

Statistical analysis was performed using Prism (version 5.03, GraphPad, La Jolla, CA, USA.). The Mann–Whitney U test was applied for the two-group comparison and one-way analysis of variance (ANOVA) for the multi-group comparison. All the results were presented as means ± standard errors. A *p* value of less than 0.05 was regarded as significant.

## 3. Results

### 3.1. Confluence-Initiated and Factor-Induced Neurogenic Differentiation Effects on Human ADSCs In Vitro

**Figure 1:**
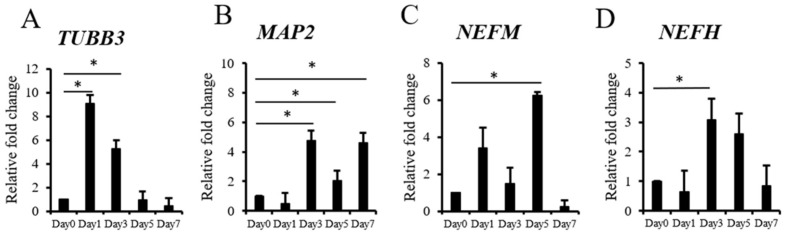
Neuron-related gene expressions of the ADSCs during confluence-initiated differentiation. The time course experiments of detecting neuron-associated gene expressions include class III β-tubulin (*TUBB3*) (**A**), microtubule-associated protein 2 (*MAP2*) (**B**), neurofilament medium polypeptide (*NEFM*) (**C**), and neurofilament heavy polypeptide (*NEFH*) (**D**) in the spontaneously differentiated ADSCs after high confluence was achieved in the cultures by RT-qPCR. The value of each gene expression was normalized to the expression of glyceraldehyde-3-phosphate dehydrogenase (*GAPDH*). Relative fold changes of the values from day 1 to day 7 were compared with the value of day 0 (without induction) as one. The values are expressed as mean ± S.D. with three experimental replicates. Statistical significance was determined using the Mann–Whitney U test. “*” represents *p* < 0.05.

Spontaneous factor-induced neurogenic differentiation was observed by detecting the neuronal marker gene expressions of the ADSC differentiated cells by RT-qPCR. Neuron-associated gene expressions, such as *TUBB3* (Figure 1A), *MAP2* (Figure 1B), *NEFM* (Figure 1C), and *NEFH* (Figure 1D), were monitored for seven days. The expressions of *TUBB3*, *MAP2*, and *NEFH* were all significantly enhanced on day 3. The *TUBB3* expression on day 1 was significantly higher than that on day 0. The *MAP2* expressions on days 5 and 7 were also significantly increased when compared with those on day 0. In addition, the expression of *NEFM* was significantly elevated on day 5. These genes were highly expressed in the confluence-initiated differentiation of the ADSCs within seven days, which indicated the neurogenic differentiation of ADSCs could spontaneously happen at high confluence.

### 3.2. Comparison of Confluence-Initiated Neurogenic Differentiation on the ADSCs and BMSCs

**Figure 2:**
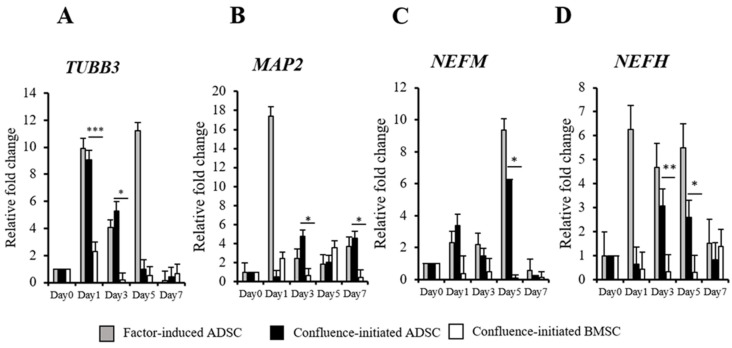
Comparison of neuron-related gene expressions of the ADSCs and BMSCs during confluence-initiated neurogenic differentiation. The time course experiments of detecting neuron-associated gene expressions include class III β-tubulin (*TUBB3*) (**A**), microtubule-associated protein 2 (*MAP2*) (**B**), neurofilament medium polypeptide (*NEFM*) (**C**), and neurofilament heavy polypeptide (*NEFH*) (**D**) in the spontaneously differentiated ADSCs after high confluence was achieved in the cultures by RT-qPCR. The value of each gene expression was normalized to the expression of glyceraldehyde-3-phosphate dehydrogenase (*GAPDH*). Relative fold changes of the values from day 1 to day 7 were compared with the value of day 0 (without induction) as one. The values are expressed as mean ± S.D. with three experimental replicates. Statistical significance to compare confluence-initiated differentiation of the ADSCs and BMSCs was determined using one-way ANOVA analysis. “*” represents *p* < 0.05. “**” represents *p* < 0.01. “***” represents *p* < 0.001.

**Figure 3:**
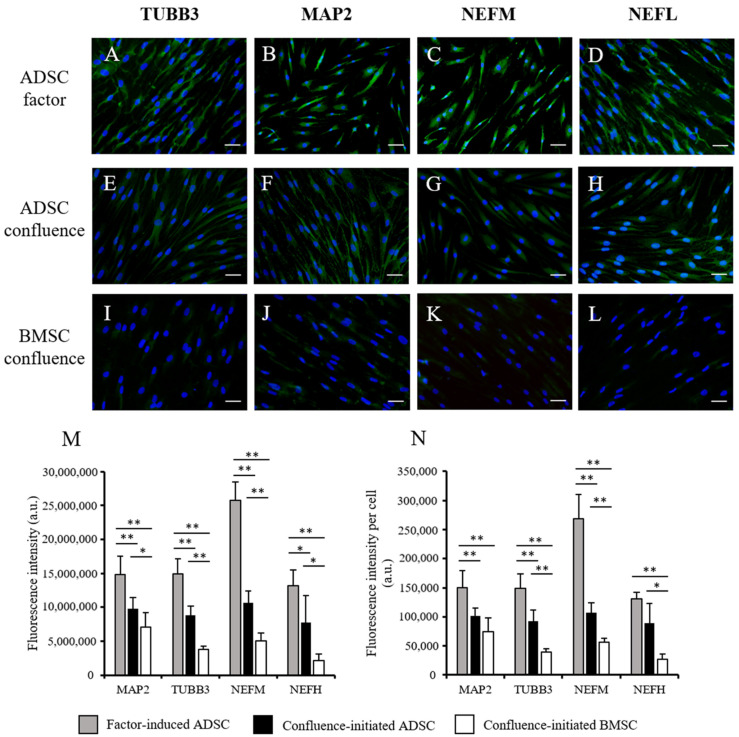
Comparison of neuron-related protein expressions of the ADSCs and BMSCs during confluence-initiated neurogenic differentiation. (**A**,**E**,**I**) Class III β-tubulin (TUBB3), (**B**,**F**,**J**) microtubule-associated protein 2 (MAP2), (**C**,**G**,**K**) neurofilament medium polypeptide (NEFM), and (**D**,**H**,**L**) neurofilament light polypeptide (NEFL) of the factor-induced ADSCs, confluence-initiated ADSCs, and confluence-initiated BMSC differentiated neuron-like cells on day 7 detected by immunofluorescence staining are shown in green fluorescence. DNA stained by DAPI is shown in blue fluorescence (magnification × 100; the scale bar = 100 µm). (**M**) The mean of the total immunofluorescence intensity from a fixed number of cells in six fields of one slide was quantified by the Image-Pro Plus v4.5.0.29. (**N**) The mean of the value that was normalized to the cell numbers in the field was calculated. All the values are expressed as mean ± S.D. with three experimental replicates. Statistical significance to compare the multiple groups was determined using one-way ANOVA analysis. “*” represents *p* < 0.05. “**” represents *p* < 0.01.

**Figure 4:**
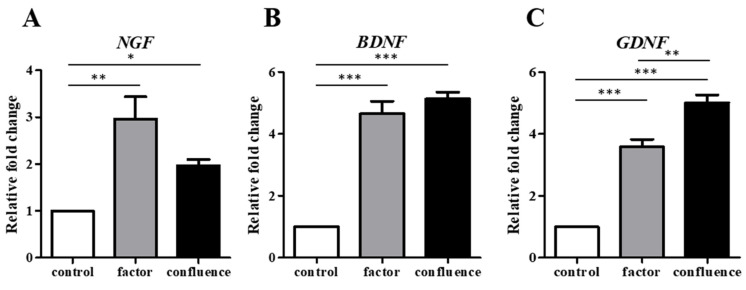
Neurotrophic gene expressions of the ADSCs during factor-induced and confluence-initiated neurogenic differentiation. Neurotrophic gene expressions, including the nerve growth factor (*NGF*) (**A**), brain-derived neurotrophic factor (*BDNF*) (**B**), and cell-derived neurotrophic factor (*GDNF*) (**C**) in the factor-induced (factor) and confluence-initiated (confluence) differentiated ADSCs on day 7 were detected by RT-qPCR. The value of each gene expression was normalized to the expression of glyceraldehyde-3-phosphate dehydrogenase (*GAPDH*). Relative fold changes of the values of the factor-induced and confluence-initiated differentiated ADSCs were compared with the value of the control (without induction) as one. The values are expressed as mean ± SEM with three experimental replicates. Statistical significance was determined using the one-way ANOVA test. “*” represents *p* < 0.05. “**” represents *p* < 0.01. “***” represents *p* < 0.001.

To determine the confluence-initiated neurogenic differentiation on the ADSCs and BMSCs, the neuron-associated gene expressions in the factor-induced and confluence-initiated neurogenic differentiation of the ADSCs and BMSCs were compared by RT-qPCR. *TUBB3* (Figure 2A), *MAP2* (Figure 2B), *NEFM* (Figure 2C), and *NEFH* (Figure 2D) were all highly expressed in the confluenced-initiated differentiated ADSCs but not in the BMSCs. The expression of *TUBB3* of the confluence-initiated differentiation of the ADSCs on days 1 and 3 was significantly higher than that of the BMSCs. *MAP2* and *NEFH* on day 3 and *NEFH* and *NEFM* on day 5 in the confluence-initiated differentiation of the ADSCs were significantly higher than that of the BMSCs. The neuron-associated protein expressions, including TUBB3 (Figure 3A), MAP2 (Figure 3B), NEFM (Figure 3C), and NEFL (Figure 3D), were detected in the factor-induced neurogenic differentiation of the ADSCs through IF analysis. These proteins were also observed to be upregulated in the confluence-initiated differentiation of the ADSCs (Figure 3E–H). However, the neurogenic differentiation of the BMSCs could not be induced by confluence since all genes (Figure 2) and proteins (Figure 3I–L) were not upregulated. According to the results of quantifying the total immunofluorescent intensity (Figure 3M) and the fluorescence of each cell (Figure 3N), the intensities of the four proteins in the confluence-initiated differentiation of the ADSCs were significantly higher than those of the BMSC group, except the result of the fluorescent intensity of the MAP2 normalized with cells. Besides, the expression of neurotrophins on day 7, including the nerve growth factor (*NGF*) (Figure 4A), brain-derived neurotrophic factor (*BDNF*) (Figure 4B), and glial cell-derived neurotrophic factor (*GDNF*) (Figure 4C), was significantly upregulated in the confluence-initiated differentiation of the ADSCs compared with that of the control group on day 0.

### 3.3. Comparison of the ADSCs and BMSCs in Confluence-Initiated Adipogenic and Osteogenic Differentiation

**Figure 5:**
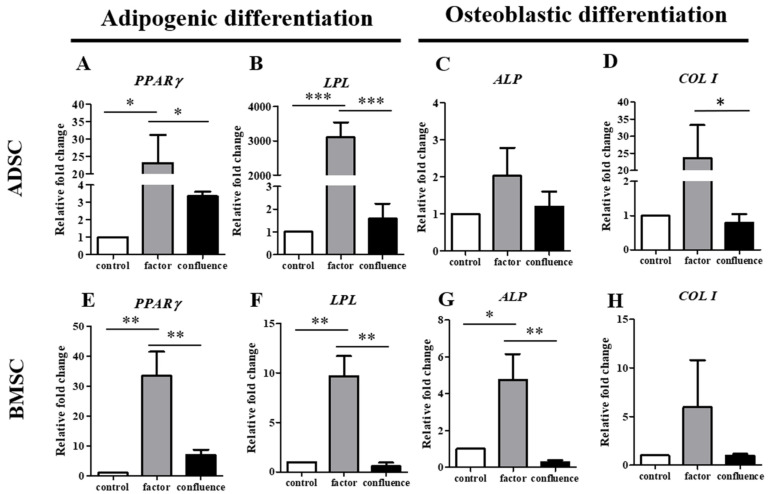
Detection of adipose-related and osteocyte-related gene expressions of the ADSCs and BMSCs during confluence-initiated differentiation. Adipose-related genes, including peroxisome proliferator-activated receptor γ (*PPARγ*) (**A**,**E**) and lipoprotein lipase (*LPL*) (**B**,**F**), and osteocyte-related genes, including alkaline phosphatase (*ALP*) (**C**,**G**) and collagen type I (*COL I*) (**D**,**H**) of the ADSCs and BMSCs, after the factor-induced (factor) and confluence-initiated (confluence) differentiation on day 7 were detected by RT-qPCR. The value of each gene expression was normalized to the expression of glyceraldehyde-3-phosphate dehydrogenase (*GAPDH*). Relative fold changes of the values of the factor-induced and confluence-initiated differentiated ADSCs were compared with the value of the control (without induction) as one. The values are expressed as mean ± SEM with three experimental replicates. Statistical significance was determined using the one-way ANOVA test. “*” represents *p* < 0.05. “**” represents *p* < 0.01. “***” represents *p* < 0.001.

The adipogenic and osteogenic differentiation in the ADSCs and BMSCs was also determined by RT-qPCR. Peroxisome proliferator-activated receptor γ (*PPARγ*) and lipoprotein lipase (*LPL*) genes were detected to verify the adipogenic differentiation in the ADSCs (Figure 5A,B) and BMSCs (Figure 5E,F). Alkaline phosphatase (*ALP*) and collagen type I (*COL I*) genes were used to determine the osteogenic differentiation of the ADSCs (Figure 5C,D) and BMSCs (Figure 5G,H). Adipogenic and osteogenic genes were highly expressed in the factor-induced differentiation of the ADSCs and BMSCs, which explained that fact that differentiation could be induced by a factor-inducing medium. However, there were significantly fewer adipogenic genes in the differentiated ADSCs and BMSCs in the confluent situation. In the confluence-initiated osteogenic differentiation, neither *ALP* nor *COL I* was high in the differentiated ADSCs and BMSCs.

## 4. Discussion

In this study, we found that the confluent ADSCs at passages 3 to 5 without additional induction would express neurogenic genes and proteins that showed the tendency toward spontaneous neurogenic differentiation, but not adipogenic and osteogenic differentiation. However, spontaneous neurogenic, adipogenic, and osteogenic differentiation of the confluent BMSCs at the same passages as the ADSCs was not observed in this study.

Stem cells have an inherent propensity to differentiate into different lineages according to their origins. Tendon-derived stem cells from tendon tissue had the potential of spontaneous tendongenic differentiation [44]. ADSCs from early passages (P1–P3) were determined to have better abilities to differentiate into adipocytes spontaneously [45]. Moreover, the potential of spontanous adipogenic, chondrogenic, and osteogenic differentiation on ADSCs maintained in a proliferation medium for 14 days without further passaging was also found [46]. However, spontaneous differentiation might not be beneficial to MSCs. MSCs from the placenta went through aging and spontaneous osteogenic differention upon routine in vitro cultative expansion, and the supplementation of the basic fibrobliast growth factor (bFGF) could enhance proliferation and suppress spontaneous differentiation. One explanation is that the physiological niches of MSCs would prevent spontaneous differentiation and promote self-renewal [47]. Extensive passaging (>passages 10) and low density seeding were reported to cause spontaneous osteogenic differentiation of ADSCs and their decreased adipogenic potential, the results of which might link to cell senescence [48]. Rat BMSCs were also found to have the tendency of spontaneous osteogenic differentiation after being cultured for 21 days without osteogenic induction [49]. However, the cultative passages of the ADSCs and BMSCs were not longer than five times, and the culture period for each passage was not longer than seven days in this study. The MSCs were still young enough to keep the stemness and prevent spontaneous differentiation. These might be the reasons why the results of this study did not exhibit spontaneous adipogenic and osteogenic differentiation of the ADSCs and BMSCs.

In this study, we found that the confluent human ADSCs at passages 3 to 5, which had been continuously cultured for seven days, had the tendency toward spontaneous neurogenic differentiation. The cause of spontaneous neurogenic differentiation in the ADSCs might be due to cell confluence, which was the reason why the differentiation was “confluence-initiated”. ADSCs cultured at high density highly expressed growth factors that are associated with differentiation [39]. In addition, ADSCs from young donors exhibited high baseline expression levels of neural and glial markers [50]. The source of the ADSCs in this study was from females under 30 years old. We suggest that spontaneous neurogenic differentiation of ADSCs from young donors could be induced by cell confluence, and without a long-term culture in vitro. One explanation suggested that MSCs had the tendency toward neurogenic differentiation due to the origin of the neuropithelium or neural crest [51]. Another reason was that the cellular stress of the in vitro artificial culture enviroment might cause spontaneous neurogenic marker expressions of MSCs [52]. However, the mechanism needs to be studied further.

MSCs can secrete trophic factors, such as hepatocyte growth factor (HGF), stromal derived factor-1 (SDF-1), insulin-like growth factor (IGF-1), epithelial growth factor (EGF), nerve growth factor (NGF), transforming growth factor-alpha (TGF-α), and tissue angiogenesis vascular endothelial growth factor (VEGF), which enhance cell survival. VEGF, TGF-β, and β-FGF, which play important roles in wound healing, contribute to angiogenesis, granulation tissue formation, and re-epithelialization [42]. The growth factors may be stimulated by the use of ADSCs. On the other hand, platelet-rich plasma (PRP) is a source of growth factors, such as IGF-1, VEGF, etc., that can also be applied to wound healing [12]. The confluence and differentiation of ADSCs may be affected when ADSCs and PRP are co-administered. Neurotrophins, including NGF, BDNF, and GDNF, help the growth and function of neurons, and they were detected in the conditioned medium of the spontaneously differentiated ADSCs to confirm the neurogenic differentiation potential in this study. In the confluent population of ADSCs, neurotrophins secreted by the spontaneous neurogenic differentiated ADSCs may easily enhance other peripheral ADSCs toward neural differentiation. However, one limitation of this study is that it is still unknown whether confluence-initiated spontaneous neurogenic differentiation could make MSCs become functional neurons or not. Further studies are needed. In a previous study, primary rat BMSCs were observed to spontaneously differentiate into neural precursor cells after a six-week culture [53]. Another study declared rat BMSCs were also found to self-differentiate into neuron-like cells, and the researchers proposed that increased NGF and the gene expressions of the receptors TrkA and TrkB in rat BMSCs without external induction might cause self-differentiation [54]. However, confluence-initiated spontaneous neurogenic differentiation of the BMSCs was not observed in this study. Due to the fact that the BMSCs used in this study were delinked from the donors, we could not reveal the correlation of donor age and spontaneous neurogenic differentiation in the BMSCs.

The spontaneous neurogenic differentation potential of ADSCs might be increased due to cellular confluence, but the lack of evidence suggests that mature neurons could be derived from spontaneous differentiated ADSCs. This study reveals the possibility of the clinical application for confluent ADSCs to be injected into a nerve conduit to treat nerve injury. Compared with BMSCs, highly confluent ADSCs may tend to exhibit spontaneous neural differentiation that benefits nerve repair.

## Figures and Tables

**Table 1:** Primer sequences used in this study for the RT-qPCR analysis.

Gene	Primer Sequence	Accession Number
*MAP2*	F: CAAACGTCATTACTTTACAACTTGA R: CAGCTGCCTCTGTGAGTGGAG	X54100.1
*TUBB3*	F: GCCAAGTTCTGGGAGGTCATC R: GTAGTAGACAACTGATGCGTTCCA	NM_139254
*NEFM*	F: AGTGGTTCAAATGCCGCTAC R: TTTTCCAGCTGCTGGATGGT	NM_017029
*NEFH*	F: AGTGGTTCCGAGTGAGATTG R: CTGCTGAATTGCATCCTGGT	NM_012607
*NGF*	F: AGCGCAGCGAGTTTTGGC R: CCGCCTGTATGCCGATCAGA	NM_002506
*BDNF*	F: AGAGGCTTGACATCATTGGCTG R: CAAAGGCACTTGACTACTGAGCATC	EF689042
*GDNF*	F: CACCAGATAAACAAATGGCAGTGC R: CGACAGGTCATCATCAAAGGCG	NM_000514
*PPARγ*	F: TCAGGTTTGGGCGGATGC R: TCAGCGGGAAGGACTTTATGTATG	NM_138712
*LPL*	F: TGTAGATTCGCCCAGTTTCAGC R: AAGTCAGAGCCAAAAGAAGCAGC	NM_000237
*ALP*	F: CGCTTGTGCCTGGACGGAC R: GGGGTCTTTCTCTTTCTCTGGCAC	MN308913
*COL I*	F: GAGGGCCAAGACGAAGACGAAGACATC R: CAGATCACGTCATCGCACAAC	NM_000088
*GAPDH*	F: CAACTCCCTCAAGATTGTCAGCAA R: GGCATGACTGTGGTCATGA	NM_017008
